# The NADPH oxidases DUOX1 and DUOX2 are sorted to the apical plasma membrane in epithelial cells via their respective maturation factors DUOXA1 and DUOXA2

**DOI:** 10.1111/gtc.13153

**Published:** 2024-08-10

**Authors:** Akira Kohda, Sachiko Kamakura, Junya Hayase, Hideki Sumimoto

**Affiliations:** ^1^ Department of Biochemistry Kyushu University Graduate School of Medical Sciences Fukuoka Japan

**Keywords:** apical membrane, apical sorting, DUOX1, DUOX2, DUOXA1, DUOXA2, epithelial cells, NADPH oxidase, NOX5

## Abstract

The membrane‐integrated NADPH oxidases DUOX1 and DUOX2 are recruited to the apical plasma membrane in epithelial cells to release hydrogen peroxide, thereby playing crucial roles in various functions such as thyroid hormone synthesis and host defense. However, it has remained unknown about the molecular mechanism for apical sorting of DUOX1 and DUOX2. Here we show that DUOX1 and DUOX2 are correctly sorted to the apical membrane via the membrane‐spanning DUOX maturation proteins DUOXA1 and DUOXA2, respectively, when co‐expressed in MDCK epithelial cells. Impairment of *N*‐glycosylation of DUOXA1 results in mistargeting of DUOX1 to the basolateral membrane. Similar to DUOX1 complexed with the glycosylation‐defective DUOXA1, the naturally non‐glycosylated oxidase NOX5, which forms a homo‐oligomer, is targeted basolaterally. On the other hand, a mutant DUOXA2 deficient in *N*‐glycosylation is less stable than the wild‐type protein but still capable of recruiting DUOX2 to the apical membrane, whereas DUOX2 is missorted to the basolateral membrane when paired with DUOXA1. These findings indicate that DUOXA2 is crucial but its *N*‐glycosylation is dispensable for DUOX2 apical recruitment; instead, its C‐terminal region seems to be involved. Thus, apical sorting of DUOX1 and DUOX2 is likely regulated in a distinct manner by their respective partners DUOXA1 and DUOXA2.

## Graphical abstract

In polarized MDCK epithelial cells, DUOX1 and DUOX2 are recruited to the apical plasma membrane when complexed with DUOXA1 and DUOXA2, respectively. Apical sorting of DUOX1 requires N‐glycosylation of DUOXA1, whereas DUOXA2 targets DUOX2 to the apical membrane in a manner independent of its N‐glycosylation but dependent on its C‐terminal cytoplasmic region.

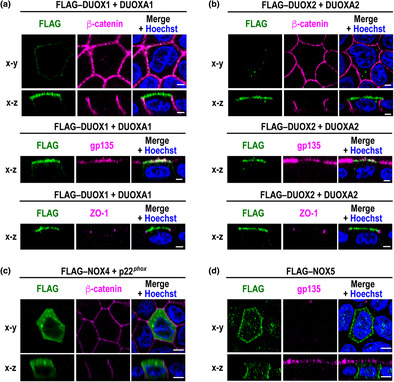

## 1 INTRODUCTION

The dual oxidases DUOX1 and DUOX2 belong to the membrane‐integrated NADPH oxidase (NOX) family, which contains another five members (NOX1–NOX5) in mammalian genomes (Lambeth, 2004; Leto et al., 2009; Sumimoto, 2008). In the polarized epithelium, DUOX1 and DUOX2 are recruited to the apical domain of the plasma membrane (PM) (El Hassani et al., 2005; Luxen et al., 2009; Morand et al., 2009), and thus release hydrogen peroxide (H_2_O_2_) to the luminal side for thyroid hormone synthesis by thyrocytes (De Deken & Miot, 2019; Moreno et al., 2002) and for host defense by digestive and bronchoalveolar epithelial cells (Forteza et al., 2005; Geiszt et al., 2003; Grasberger et al., 2021; Pircalabioru et al., 2016); DUOX2 is the primary DUOX in thyrocytes and enterocytes, while airway epithelial cells predominantly express DUOX1. Apical sorting of DUOX1 and DUOX2 is crucial for their functions, because thyroid hormone synthesis, an event requiring H_2_O_2_, occurs at the interface between the apical PM and the colloid‐filled lumen; and the reactive oxidant H_2_O_2_ likely contributes to host defense at the apical side of surface epithelia exposed to microorganisms. However, little is known how these oxidases are sorted to the apical PM.

In polarized epithelial cells, transmembrane proteins synthesized at the endoplasmic reticulum (ER) are transported to the Golgi apparatus and sorted from the *trans*‐Golgi network to the apical or basolateral PM (Rodriguez‐Boulan & Macara, 2014). The ER‐to‐Golgi transport and subsequent PM delivery of DUOX1 and DUOX2 require physical interactions with their respective maturation factors DUOXA1 and DUOXA2, both of which are *N‐*glycosylated proteins with five transmembrane segments (TM1–TM5) (Grasberger & Refetoff, 2006; Luxen et al., 2009; Morand et al., 2009). However, it has remained unknown about the role for the DUOXA proteins in apical sorting of DUOX1 and DUOX2. The DUOX oxidases also undergo *N*‐linked glycosylation in the ER (De Deken et al., 2002; Morand et al., 2003), which is essential for PM targeting of the DUOX–DUOXA complexes in HEK 293 cells (Ueyama et al., 2015). In addition, defective *N*‐glycosylation of DUOXA1 leads to an only slight decrease in cell surface expression of DUOX1 in COS‐7 cells, but PM delivery of DUOX2 is drastically impaired when co‐expressed with a glycosylation‐defective mutant of DUOXA2 (Poncelet et al., 2019). The role for *N*‐glycosylation in DUOX localization, however, has not been tested in polarized cells. Whereas extracellular *N*‐glycans occasionally play a role in protein folding and stabilization as well as ER‐to‐Golgi transport and subsequent delivery to PM (Ellgaard et al., 2016; Esmail & Manolson, 2021), they can also function as an apical sorting signal (Rodriguez‐Boulan & Macara, 2014; Stoops & Caplan, 2014).

In the present study, we have used polarized MDCK epithelial cells to test the role for DUOXA1 and DUOXA2, with special attention to their *N*‐glycosylation, in apical sorting of the DUOX oxidases. DUOXA1 recruits DUOX1 to the apical PM in a manner dependent on its *N*‐glycosylation. On the other hand, DUOXA2 is crucial but its *N*‐glycosylation is dispensable for apical targeting of DUOX2, although this modification stabilizes DUOXA2 at the protein level. Instead, the C‐terminal cytoplasmic region of DUOXA2 seems to participate in proper sorting of DUOX2.

## 2 RESULTS AND DISCUSSION

### 2.1 DUOX1 and DUOX2 but not NOX4 or NOX5 are delivered to the apical PM in MDCK cells

**FIGURE 1:**
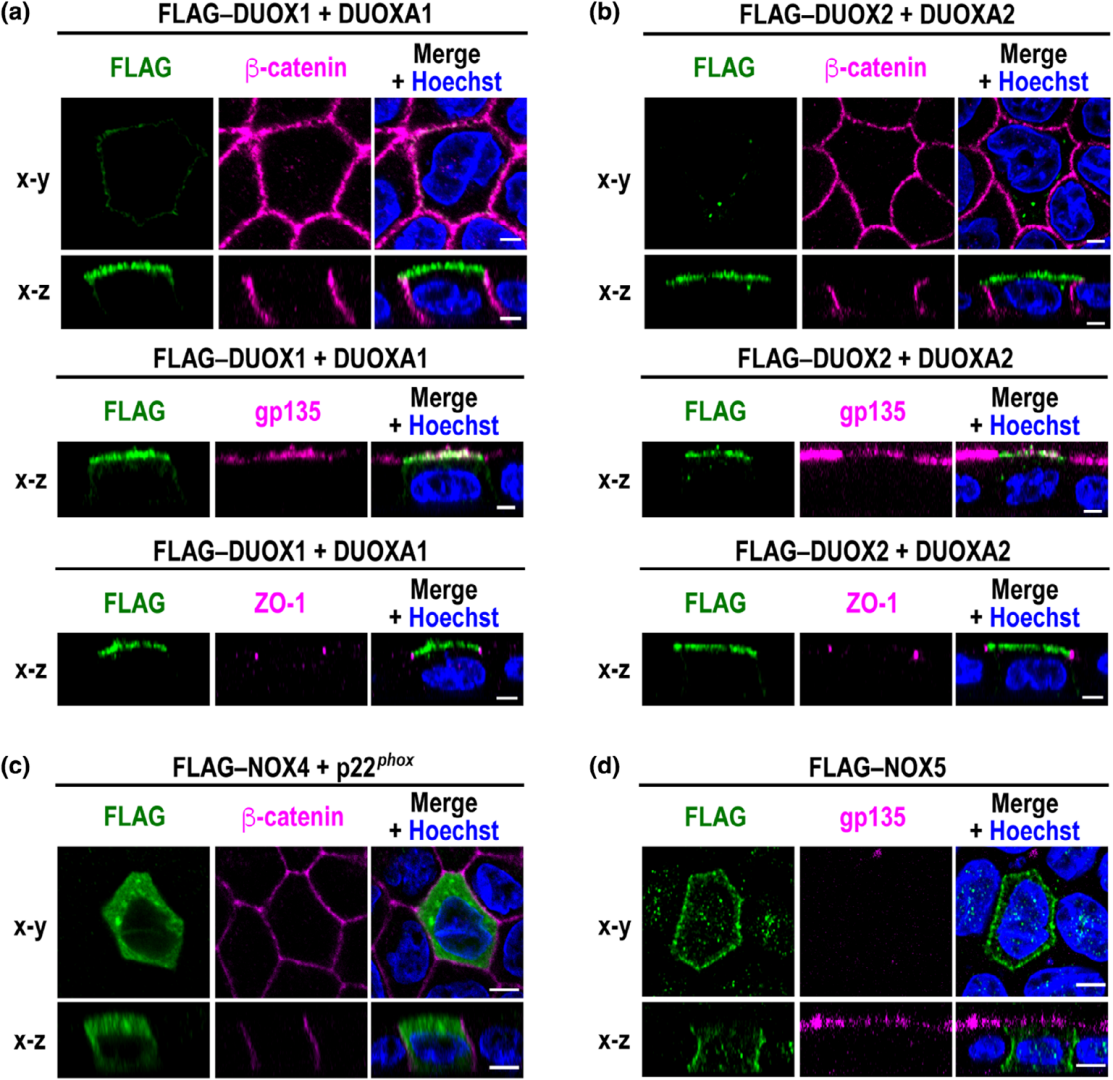
Intracellular recruitment of DUOX1–DUOXA1, DUOX2–DUOXA2, NOX4‐p22^*phox*^, and NOX5 in MDCK canine kidney epithelial cells. Shown are representative confocal images of MDCK cells expressing FLAG–DUOX1 and DUOXA1 (a), FLAG–DUOX2 and DUOXA2 (b), FLAG–NOX4 and p22^*phox*^ (c), or FLAG–NOX5 (d). Cells were co‐stained with the anti‐FLAG antibody (Ab), the nuclear marker Hoechst, and the Ab for the apical glycoprotein gp135 (podocalyxin), the basolateral marker β‐catenin, or the tight junction protein ZO‐1. Horizontal (*x*–*y*) sections are shown at the level of middle (lateral) planes, and *x*–*z* images of a vertical section are taken at the midpoint of the image. Scale bars, 5 μm.

To study the localization of DUOX1 in MDCK canine kidney epithelial cells, we co‐expressed FLAG–DUOX1 and its maturation factor DUOXA1. As visualized by confocal laser microscopy (Figure 1a), FLAG–DUOX1 colocalized with the apical glycoprotein gp135 but not with the basolateral marker β‐catenin or with the tight junction protein ZO‐1 in MDCK cells. Similarly, FLAG–DUOX2 was recruited to the apical surface in the presence of its partner DUOXA2 (Figure 1b). Thus, the NOX family oxidases DUOX1 and DUOX2 appear to be specifically sorted to the apical PM in polarized MDCK cells.

NOX4 is known to form a heterodimer with the membrane‐spanning protein p22^*phox*^ to localize to several intracellular sites, especially the ER, in various types of cells (Prior et al., 2016; von Löhneysen et al., 2008; Zana et al., 2018). In polarized MDCK cells, FLAG*–*NOX4 was also distributed in the cytoplasm when co‐expressed with p22^*phox*^ (Figure 1c). On the other hand, NOX5, which forms an active homo‐oligomer but does not associate with other transmembrane proteins including p22^*phox*^ (Kawahara et al., 2011), was mainly delivered to the basolateral PM (Figure 1d). Thus, NOX family members seem to be specifically targeted to distinct membranes in polarized epithelial cells.

### 2.2 Apical sorting of DOUX1 depends on *N*‐glycosylation of DUOXA1

**FIGURE 2:**
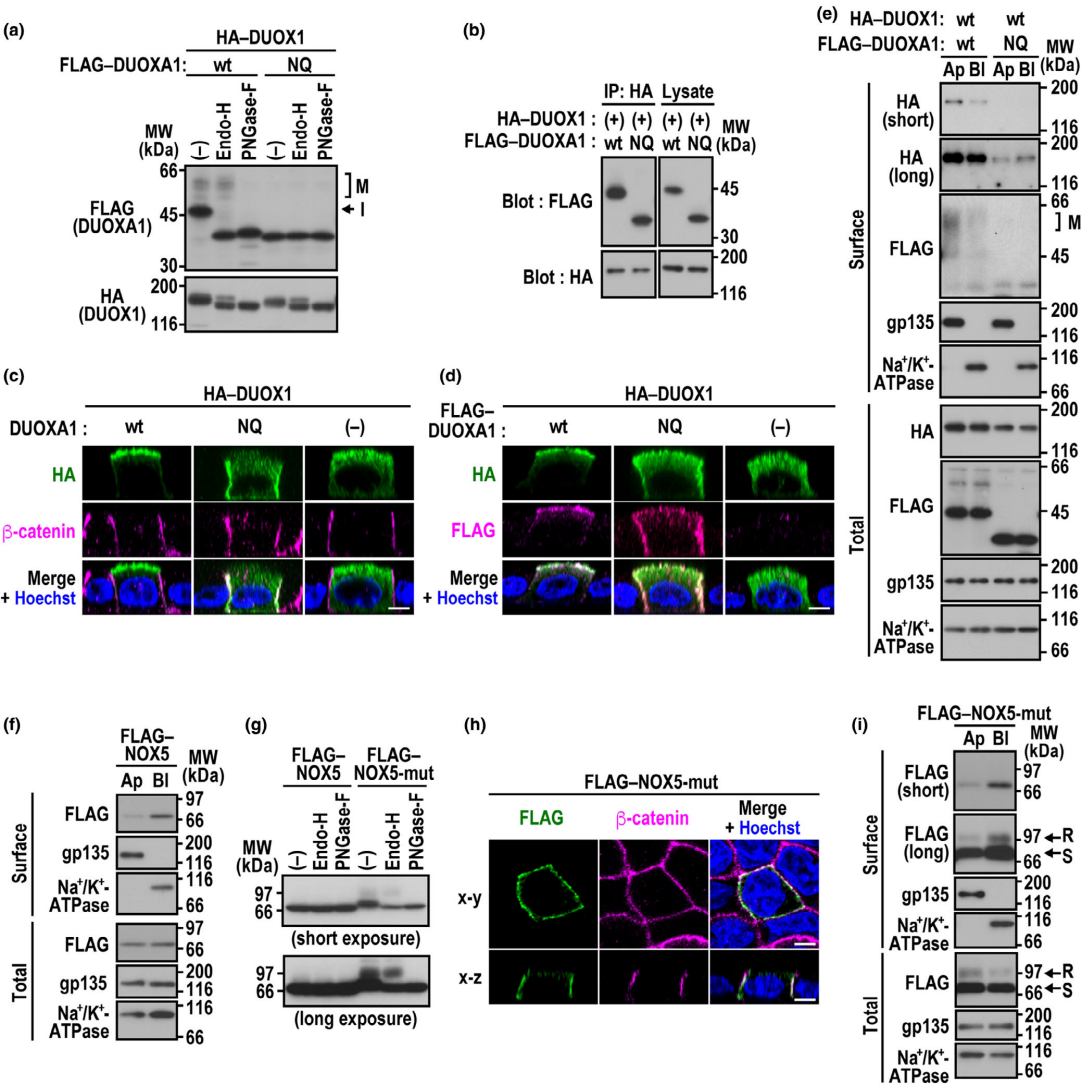
Role for *N*‐glycosylation in apical sorting of DUOX1 and basolateral targeting of NOX5. (a) *N*‐glycosylation of DUOXA1. MDCK cells expressing HA–DUOX1 and wild‐type (wt) DUOXA1 or the mutant DUOXA1 (NQ) with the N84Q/N109Q/N121Q substitution as a FLAG‐tagged protein were lysed with Triton X‐100. The extracted proteins were digested with Endo‐H or PNGase‐F, followed by immunoblot analysis with the anti‐FLAG or anti‐HA antibody (Ab). Proteins with Endo‐H‐resistant, mature *N*‐glycans (M) and those with Endo‐H‐sensitive, immature *N*‐glycans (I) are indicated. (b) Role for *N*‐glycosylation of DUOXA1 in its interaction with DUOX1. Proteins in the cell lysate from HEK293 cells co‐expressing FLAG–DUOXA1 (wt) or FLAG–DUOXA1 (NQ) with HA–DUOX1 were immunoprecipitated (IP) with the anti‐HA Ab or control IgG, followed by immunoblot analysis (Blot) with the anti‐FLAG or anti‐HA Ab. (c), (d) Role for *N*‐glycosylation of DUOXA1 in apical sorting of the DUOX1–DUOXA1 complex, as shown by confocal microscopy. MDCK cells expressing HA–DUOX1 with DUOXA1 (wt) or DUOXA1 (NQ) (c) or those expressing HA–DUOX1 with FLAG–DUOXA1 (wt) or FLAG–DUOXA1 (NQ) (d) were stained with the anti‐FLAG and/or anti‐HA Ab and with the Ab for the basolateral marker β‐catenin and the nucleus marker Hoechst. Representative *x*–*z* images of a vertical section are shown. (e) Role for *N*‐glycosylation of DUOXA1 in apical sorting of the DUOX1–DUOXA1 complex, as shown by domain‐selective cell surface biotinylation. Proteins at the apical (Ap) or basolateral (Bl) surfaces in MDCK cells expressing HA–DUOX1 and FLAG–DUOXA1 (wt) or FLAG–DUOXA1 (NQ) were biotinylated, and the biotinylated proteins were precipitated with streptavidin‐Sepharose beads and analyzed by immunoblot with the anti‐HA Ab, the anti‐FLAG Ab, the Ab for the apical transmembrane protein gp135, or the Ab for the basolateral membrane‐spanning protein Na^+^/K^+^‐ATPase. Shorter (short) and longer (long) exposures of the same blot are shown. (f), (i) Basolateral sorting of NOX5. Proteins at the apical (Ap) or basolateral (Bl) surfaces in MDCK cells expressing NOX5 (wt) (f) or the glycosylated mutant of NOX5 (NOX5‐mut) (i) as a FLAG‐tagged protein were subjected to domain‐selective cell surface biotinylation analysis as in (e). Proteins with Endo‐H‐resistant *N*‐glycans (R) and those with Endo‐H‐sensitive *N*‐glycans (S) are indicated. (g) *N*‐glycosylation of NOX5‐mut. The lysate from MDCK cells expressing FLAG–NOX5 (wt) or FLAG–NOX5‐mut were digested with Endo‐H or PNGase‐F, followed by immunoblot analysis with the anti‐FLAG Ab. (h) Basolateral sorting of NOX5‐mut. MDCK cells expressing FLAG–NOX5‐mut were stained with the anti‐FLAG Ab, the anti‐β‐catenin Ab, and Hoechst. A representative horizontal (*x*–*y*) section is shown at the level of a middle plane, and the *x*–*z* image of a vertical section is taken at the midpoint of the image. Positions for marker proteins are indicated in kilodaltons (kDa). Scale bars, 5 μm.

Because *N*‐glycans function as an apical sorting signal for several protein complexes (Rodriguez‐Boulan & Macara, 2014; Stoops & Caplan, 2014), we tested the role for *N*‐glycosylation of DUOXA1 in apical sorting of the DUOX1–DUOXA1 complex. In MDCK cells, FLAG–DUOXA1 paired with HA–DUOX1 was expressed as two bands at about 45 and 50 kDa on SDS‐PAGE (Figure 2a). The 45‐kDa protein was sensitive to treatment with peptide: *N*‐glycosidase F (PNGase‐F) and with endoglycosidase H (Endo‐H), suggestive of its identity as an immature form with high‐mannose‐type *N*‐glycans; the 50‐kDa DUOXA1 was resistant to Endo‐H, indicating its passage via the Golgi apparatus as a mature form with complex‐type *N*‐glycans. Thus, DUOXA1 indeed undergoes *N*‐glycosylation in MDCK cells.

DUOXA1 has four putative sites for *N*‐glycosylation (NxS/T): N71 is located in TM2 and thus unlikely modified; on the other hand, N84, N109, and N121 are present in the extracellular loop between TM2 and TM3 and indeed shown to be glycosylated (Sun, 2020; Wu et al., 2021). To test the role of *N*‐glycosylation, we simultaneously replaced N84, N109, and N121 by a glutamine to construct the non‐glycosylated form of DUOXA1 (N84Q/N109Q/N121Q). As shown in Figure 2b, elimination of DUOXA1 *N*‐glycosylation did not exert a significant effect on the expression level of this protein, indicating that this modification is not involved in the stabilization of DUOXA1. Immunoblot analysis of proteins precipitated with the anti‐HA antibody revealed that FLAG–DUOXA1 (N84Q/N109Q/N121Q) fully interacted with HA–DUOX1 (Figure 2b). The glycosylation‐defective DUOXA1 and wild‐type DUOX1 were both targeted to the basolateral PM but not to the apical PM, as shown by confocal microscopic analysis (Figure 2c, d).

To further analyze the *N*‐glycosylation‐dependent apical domain sorting of the DUOX1–DUOXA1 complex, we performed domain‐selective cell surface biotinylation analysis. DUOX1 as well as the mature, complex *N*‐glycan form of wild‐type DUOXA1 was effectively delivered to the apical PM (Figure 2e). By contrast, co‐expression with the mutant DUOXA1 (N84Q/N109Q/N121Q) resulted in an impaired delivery of DUOX1 to the cell surface (Figure 2e), suggesting that *N*‐glycosylation of DUOXA1 enhances the ER‐to‐Golgi transport and subsequent PM delivery of DUOX1. Under the conditions, DOUX1 was targeted to the basolateral PM rather than the apical PM (Figure 2e), which is consistent with the results obtained by immuno‐staining analysis (Figure 2c, d). Thus, apical sorting of DOUX1 appears to require *N*‐glycosylation of its partner protein DUOXA1.

### 2.3 Basolateral sorting of NOX5 is independent of its *N*‐glycosylation

Similar to DUOX1 paired with the glycosylation‐defective DUOXA1 (N84Q/N109Q/N121Q), the confocal microscopic analysis shows that the *bona* *fide* nonglycosylated oxidase NOX5 is also delivered to the basolateral PM in MDCK cells (Figure 1d), which delivery was verified by the domain‐specific cell surface biotinylation assay (Figure 2f). In contrast to the basolateral delivery in polarized MDCK cells, Yu et al. (2014) have reported that NOX5 is localized to the apical PM in cultured human renal proximal tubular cells from hypertensive subjects but not in those from normotensive subjects, which localization was analyzed solely by cell surface biotinylation but not by confocal microscopy. The reason for this discrepancy is presently unknown; it might be due to the difference in the cell type and/or cell status.

To test whether introduction of *N*‐glycosylation affects NOX5 sorting, we constructed a mutant NOX5 with two *N*‐glycosylation sites (NOX5‐mut; see “Experimental Procedures”), which was indeed glycosylated in MDCK cells (Figure 2g). The *N*‐glycosylated NOX5‐mut, with ether Endo‐H‐resistant or ‐sensitive sugar chains, was recruited to the basolateral PM (Figure 2h, i), suggesting that NOX5 sorting is independent of *N*‐glycosylation in polarized epithelial cells.

### 2.4 Apical sorting of DOUX2 requires DUOXA2 but not its *N*‐glycosylation

**FIGURE 3:**
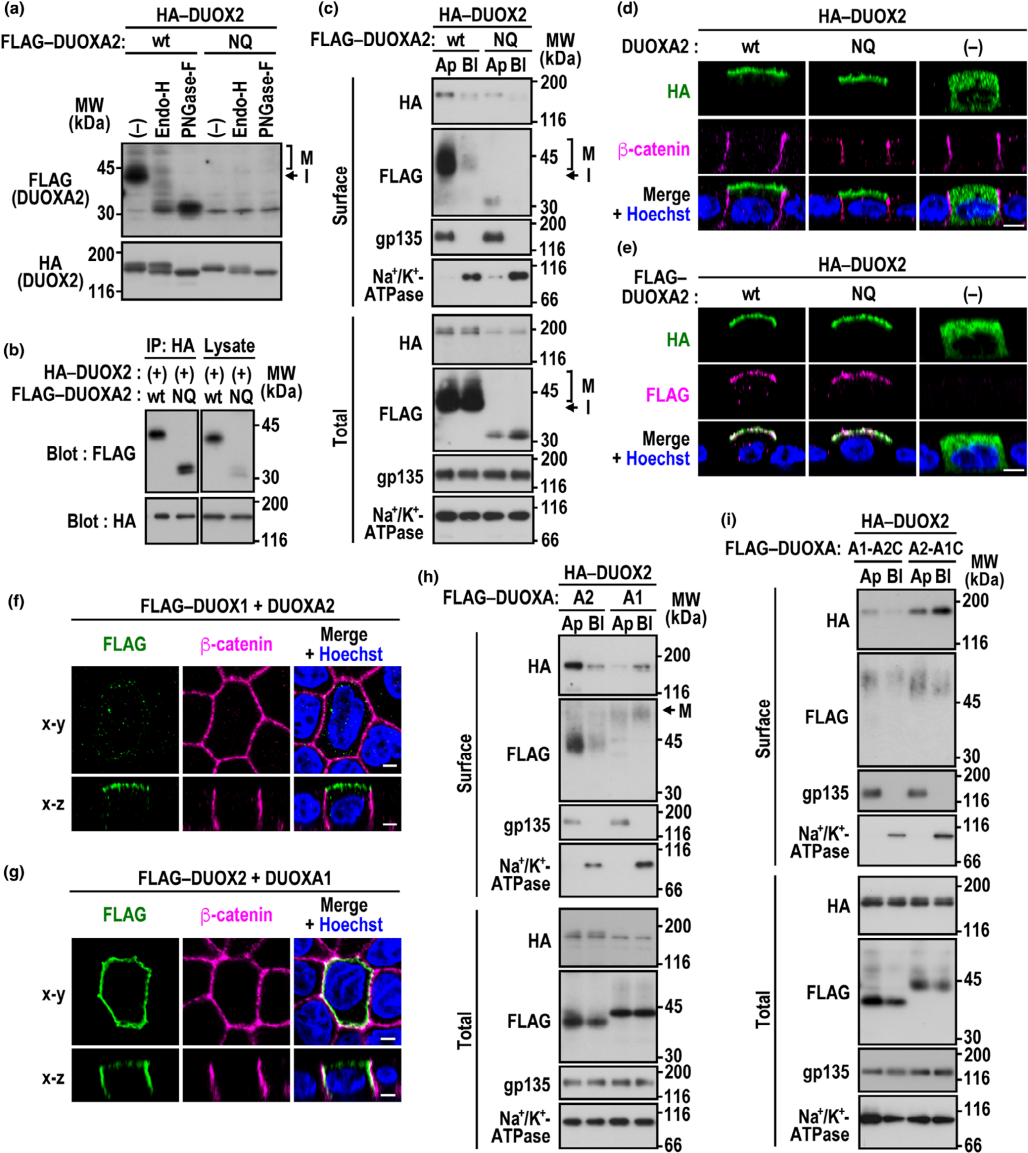
DUOXA2‐dependent apical sorting of DOUX2. (a) *N*‐glycosylation of DUOXA2. MDCK cells expressing HA–DUOX2 and wild‐type (wt) DUOXA2 or the mutant DUOXA2 (NQ) with the N84Q/N109Q/N121Q substitution as a FLAG‐tagged protein were lysed with Triton X‐100. The extracted proteins were digested with Endo‐H or PNGase‐F, followed by immunoblot analysis with the anti‐FLAG or anti‐HA antibody (Ab). Proteins with Endo‐H‐resistant, mature *N*‐glycans (M) and those with Endo‐H‐sensitive, immature *N*‐glycans (I) are indicated. (b) Role for *N*‐glycosylation of DUOXA2 in its interaction with DUOX2. Proteins in the cell lysate from HEK293 cells co‐expressing FLAG–DUOXA2 (wt) or FLAG–DUOXA2 (NQ) with HA–DUOX2 were immunoprecipitated (IP) with the anti‐HA Ab or control IgG, followed by immunoblot analysis (Blot) with the anti‐FLAG or anti‐HA Ab. (c) Role for *N*‐glycosylation of DUOXA2 in apical sorting of the DUOX2–DUOXA2 complex, as shown by domain‐selective cell surface biotinylation. Proteins at the apical (Ap) or basolateral (Bl) surfaces in MDCK cells expressing HA–DUOX2 and FLAG–DUOXA2 (wt) or FLAG–DUOXA2 (NQ) were biotinylated, and the biotinylated proteins were precipitated with streptavidin‐Sepharose beads and analyzed by immunoblot with the anti‐HA Ab, the anti‐FLAG Ab, or the Ab for the apical transmembrane protein gp135 or the Ab for the basolateral membrane‐spanning protein Na^+^/K^+^‐ATPase. (d), (e) Role for *N*‐glycosylation of DUOXA2 in apical sorting of the DUOX2–DUOXA2 complex, as shown by confocal microscopy. MDCK cells expressing HA–DUOX2 with DUOXA2 (wt) or DUOXA2 (NQ) (d) or those expressing HA–DUOX2 with FLAG–DUOXA2 (wt) or FLAG–DUOXA2 (NQ) (e) were stained with the anti‐FLAG and/or anti‐HA Ab and with the Ab for the basolateral marker β‐catenin and the nucleus marker Hoechst. Representative *x*–*z* images of a vertical section are shown. (f), (g) Apical or basolateral sorting of the mispaired DUOX–DUOXA complex. MDCK cells expressing FLAG–DUOX1 and DUOXA2 (f) or those expressing FLAG–DUOX2 and DUOXA1 (g) were stained with the anti‐FLAG Ab, the anti‐β‐catenin Ab, and Hoechst. Representative horizontal (*x*–*y*) sections are shown at the level of a middle plane, and the *x*–*z* images of a vertical section are taken at the midpoint of the image. (h) Subcellular sorting of the DUOX2–DUOXA1 complex. Proteins at the apical (Ap) or basolateral (Bl) surfaces in MDCK cells expressing HA–DUOX2 and FLAG–DUOXA1 or FLAG–DUOXA2 were subjected to domain‐selective cell surface biotinylation analysis as in (c). (i) Subcellular sorting of DUOX2 complexed with C‐terminally chimeric DUOXA proteins. Proteins at the apical (Ap) or basolateral (Bl) surfaces in MDCK cells expressing HA–DUOX2 and the FLAG‐tagged, mutant DUOXA1 with the C‐terminal region of DUOXA2 (A1‐A2C) or the FLAG‐tagged, mutant DUOXA2 with the C‐terminal region of DUOXA1 (A2‐A1C) were subjected to domain‐selective cell surface biotinylation analysis as in (c). Positions for marker proteins are indicated in kilodaltons (kDa). Scale bars, 5 μm.

As did DUOXA1 (Figure 2a), DUOXA2 underwent Golgi‐based carbohydrate modifications (Figure 3a). DUOXA2 contained three putative *N*‐glycosylation sites, N84, N109, and N121, all of which located in the extracellular loop between TM2 and TM3. The glycosylation‐defective DUOXA2 (N84Q/N109Q/N121Q) was expressed at the protein level much less than the wild‐type protein, although it was capable of binding to DUOX2 (Figure 3b). Thus, *N*‐glycosylation of DUOXA2 seems to be required for the stabilization of this protein but not for its interaction with DUOX2. Although cell surface delivery of DUOX2 was reduced by a loss of DUOXA2 *N*‐glycosylation, the DUOX2 complex was still targeted to the apical PM but not to the basolateral domain (Figure 3c). The DUOX2–DUOXA2 (N84Q/N109Q/N121Q) complex was also sorted to the apical PM, as observed by confocal microscopic analysis (Figure 3d, e). These findings indicate that *N*‐glycosylation of DUOXA2 is dispensable for apical sorting of DUOX2.

The gene pairs *DUOX1*/*DUOXA1* and *DUOX2*/*DUOXA2* each form a tightly‐linked transcriptional unit to function together (Grasberger & Refetoff, 2006). We next examined PM domain sorting of mispaired DUOX–DUOXA complexes that can be reconstituted as a partially active enzyme (Luxen et al., 2009; Morand et al., 2009). As shown in Figure 3f, DUOXA2 targeted DUOX1 to the apical PM in polarized MDCK cells. In contrast, DUOXA1 recruited DUOX2 to the basolateral PM (Figure 3g, h). The DUOXA1–DUOX2 complex is likely transported from the ER to the Golgi apparatus and then delivered to the basolateral PM since DUOXA1 on the basolateral surface was the 50‐kDa mature form with the complex‐type (i.e., Golgi‐modified) *N*‐glycans (Figure 3h). Thus, DUOXA2 appears to be specifically required for apical sorting of DUOX2. The specificity may be derived at least partially from the difference in the C‐terminal cytoplasmic region, which is not conserved between DUOXA1 and DUOXA2 (Hoste et al., 2012). This is because a mutant DUOXA2 with the C‐terminal region of DUOXA1 (DUOXA2‐A1C) was partially recruited to the lateral PM; inversely, a mutant DUOXA1 with the C‐terminal region of DUOXA2 (DUOXA1‐A2C) was rather sorted to the apical PM (Figure 3i).

### 2.5 Concluding remarks

Although apical surface localization of DUOX1 and DUOX2 is crucial for their functions, it has remained unknown about the molecular mechanism for apical sorting of these oxidases, including the role of their respective partner proteins DUOXA1 and DUOXA2. The present study shows that DUOX1 is sorted to the apical PM in a manner dependent on *N*‐glycosylation of DUOXA1. Apical sorting of DUOX2 specifically requires DUOXA2, the C‐terminal region of which appears to be involved; on the other hand, *N*‐glycosylation of DUOXA2 participates in its stability but not in proper targeting of DUOX2. Thus, apical sorting of DUOX1 and DUOX2 is likely regulated in a distinct manner by their respective partners DUOXA1 and DUOXA2. In addition, the naturally non‐glycosylated oxidase NOX5 is delivered to the basolateral PM, which delivery is not affected by introduction of *N*‐glycosylation. Thus, *N*‐glycans likely exert various effects on the polarized sorting of the NADPH oxidases DUOX1, DUOX2, and NOX5.

## 3 EXPERIMENTAL PROCEDURES

### 3.1 Plasmids and antibodies (Abs)

The cDNAs for human DUOX1 (amino acids (aa) 1‐1551) (De Deken et al., 2000; GenBank™ accession # AF230495), DUOX2 (aa 1‐1548) (De Deken et al., 2000; GenBank™ accession # AF230496), DUOXA1 (aa 1‐343) (Grasberger & Refetoff, 2006; GenBank™ accession # DQ489735), and DUOXA2 (aa 1‐320) (Grasberger & Refetoff, 2006; GenBank™ accession # DQ489734) were obtained by PCR using Human Multiple Tissue cDNA panel (BD Biosciences). The cDNAs encoding human NOX4 (aa 1‐578) (Shiose et al., 2001; GenBank™ accession # AB041035) and NOX5 (aa 1‐719) (Bánfi et al., 2001; GenBank™ accession # AF325189) were prepared, as previously described (Kiyohara et al., 2018; Matsunaga et al., 2024). The mutant NOX5 with two *N*‐glycosylation sites (NOX5‐mut) was constructed both by introducing the amino acid substitution A290N/P292T in the second extracellular loop and by inserting the mouse NOX2 fragment (aa 36‐45) that contains an *N*‐glycosylation site (N40‐Y41‐T42) into the corresponding first extracellular loop in human NOX5. The mutant DUOXA1 (DUOXA1‐A2C) was constructed by replacement of its own C‐terminus (aa 275‐343) with the DUOXA2 C‐terminus (aa 275‐320); the mutant DUOXA2 (DUOXA2‐A1C) was constructed by replacement of its own C‐terminus (aa 275‐320) with the DUOXA1 C‐terminus (aa 275‐343). Mutations leading to the indicated amino acid substitutions in DUOXA1 and DUOXA2 were introduced by PCR‐mediated site‐directed mutagenesis. The cDNA fragments were ligated to the expression vectors pcDNA3 (Invitrogen) or pEF‐BOS (Kohda et al., 2016). For expression as an N‐terminally tagged DUOX protein, DUOX1 (aa 24‐1551) and DUOX2 (aa 28‐1548) were ligated to pCMV‐8 (Sigma‐Aldrich), which was modified to contain the N‐terminal FLAG‐ or HA‐tag guided by the signal peptide sequence of preprotrypsin (Kamakura et al., 2024). All the constructs were sequenced for confirmation of their identities.

The anti‐gp135 (3F2) mouse monoclonal Ab (mAb) was generously gifted from G. K. Ojakian (State University of New York, USA). Anti‐β‐catenin rabbit polyclonal Abs (pAbs) (H‐102; #sc‐7199) were purchased from Santa Cruz Biotechnology; anti‐ZO‐1 rabbit pAbs (#61‐7300) from Thermo Fisher Scientific; the anti‐Na^+^/K^+^‐ATPase α1 subunit (EP1845Y; #ab76020) rabbit mAb form abcam; the anti‐DYKDDDDK tag mouse mAb (1E6; #014‐22,383) from FUJIFILM Wako; anti‐FLAG rabbit pAbs (#F7425) from Sigma‐Aldrich; anti‐HA mAb (16B12; #MMS‐101R) from Covance; and the mouse negative control IgG1 (#X0931) from Dako Cytomation.

### 3.2 Cell culture

MDCKII cells were cultured in Eagle's minimal essential medium with 10% fetal bovine serum (FBS). HEK293T cells were maintained in Dulbecco's modified Eagle's medium with 10% FBS.

### 3.3 Immunofluorescence microscopy

Immunofluorescence microscopy was performed as previously described (Hayase et al., 2013; Kamakura et al., 2024). MDCKII cells were transfected with the indicated cDNAs using Xtreme‐GENE HP DNA Transfection Reagent (Sigma‐Aldrich) and cultured for 4 days on a 12‐mm Transwell filter (0.4 μm pore size; Corning). The cells were then fixed for 20 min in 3.7% formaldehyde and permeabilized for 30 min with 0.5% Triton X‐100 in phosphate‐buffered saline (137 mM NaCl, 2.68 mM KCl, 8.1 mM Na_2_HPO_4_, and 1.47 mM KH_2_PO_4_, pH 7.4) containing 3% BSA. Indirect immunofluorescence analysis was performed using Alexa Fluor 488‐ or Alexa Fluor 594‐labeled goat secondary Abs (Thermo Fisher Scientific). Nuclei were stained with Hoechst 33342 (Thermo Fisher Scientific). Confocal images were captured at room temperature on the confocal microscope LSM700 (Carl Zeiss) equipped with a Plan‐Apochromat 63×/1.4 NA oil‐immersion objective lens and analyzed using ZEN (Carl Zeiss).

### 3.4 Domain‐selective cell surface biotinylation

The domain‐selective cell surface biotinylation was carried out as previously described (Sakurai et al., 2022) with minor modifications. Briefly, MDCKII cells transfected with the indicated cDNAs were cultured for 2 days on a 24‐mm Transwell filter (0.4 μm pore size; Corning). Proteins at the apical or basolateral surfaces were biotinylated by the addition of 0.5 mM EZ‐link Sulfo‐NHS‐SS‐biotin (Pierce) into the upper or lower reservoir, respectively. Cells were then lysed with the lysis buffer (2% Triton X‐100, 150 mM NaCl, and 50 mM Tris‐HCl, pH 7.5) containing Protease Inhibitor Cocktail (Sigma‐Aldrich), and biotinylated proteins were precipitated with streptavidin‐Sepharose (GE Healthcare Biosciences). For analysis of NOX5, the precipitants were washed three times with the lysis buffer; for analysis of DUOX/DUOXA, they were washed twice with 2% SDS, once with the buffer 1 (500 mM NaCl, 0.1% deoxycholate, 1% Triton X‐100, 1 mM EDTA, 50 mM Tris‐HCl, pH 7.5), once with the buffer 2 (250 mM LiCl, 0.5% deoxycholate, 0.5% NP‐40, 1 mM EDTA, 10 mM Tris‐HCl, pH 8.0), and twice with the buffer 3 (50 mM NaCl, 50 mM Tris‐HCl, pH 7.5). The washed precipitants were analyzed by immunoblot.

### 3.5 Immunoprecipitation

HEK293T cells were transfected with the indicated cDNAs and cultured for 48 h. Cells were then lysed in 0.5% Triton X‐100, 150 mM NaCl, 1 mM DTT, 5 mM EDTA, 10% glycerol, and 50 mM Tris‐HCl, pH 7.5, containing Protease Inhibitor Cocktail. Proteins in the cell lysate were immunoprecipitated using the anti‐HA (16B12) Ab or the control IgG1, coupled to protein G‐Sepharose (GE Healthcare), and the precipitants were subjected to immunoblot analysis (Kohda et al., 2016).

### 3.6 Glycosidase treatment

MDCKII cells expressing the indicated proteins were lysed in the lysis buffer containing Protease Inhibitor Cocktail. The lysate was centrifuged for 20 min at 20,000 × *g*, and the supernatant was treated with PNGase‐F (New England Biolabs) or Endo‐H (New England Biolabs), followed by immunoblot analysis.

## CONFLICT OF INTEREST STATEMENT

The authors declare no conflicts of interest.
